# MicroRNA analysis of porcine muscle tissue involved in phosphoinositol metabolism

**DOI:** 10.3389/fvets.2025.1482031

**Published:** 2025-07-23

**Authors:** Yuxiao Xie, Wenjie Cheng, Meilin Hao, Lan-lan Yi, Jun-hong Zhu, Yan-guang Zhao, Sumei Zhao

**Affiliations:** ^1^Yunnan Key Laboratory of Animal Nutrition and Feed Science, Yunnan Agricultural University, Kunming, China; ^2^College of Biology and Agriculture (College of Food Science and Technology), Zunyi Normal College, Zunyi, China; ^3^Shanghai Laboratory Animal Research Center, Shanghai, China

**Keywords:** longissimus dorsi muscle (LDM), microRNA, phosphoinositol metabolism, Diannan small ears pigs, Landrace pigs

## Abstract

**Introduction:**

To elucidate the role of miRNAs in porcine muscle tissues, this study used Solexa high-throughput sequencing to identify and compare differentially expressed miRNAs in Landrace pigs and the Diannan small ear pigs.

**Methods:**

Small RNA libraries were constructed from high-quality RNAs extracted from the Longissimus dorsi muscle (LDM) muscles of the two breeds of pigs and high-throughput sequencing was performed to identify and compare differentially expressed of miRNAs (DEmiRNAs). The target genes corresponding to the differentially expressed miRNAs were subjected to functional annotation, Gene Ontology (GO) enrichment analysis and Kyoto Encyclopedia of Genomes (KEGG) pathway enrichment analysis.

**Results:**

241 known conserved miRNAs sequences and 20 novel miRNAs in the Diannan small ear pigs library and known conserved miRNAs sequences and 22 novel miRNAs in the Landrace pigs library were identified. It is noteworthy that 14 miRNAs showed significant expression differences between the two pig breeds, and the expression levels of these miRNAs were lower in the Diannan small ear pigs than the Landrace pigs, among which the most significantly difference was miR-27a. GO and KEGG analyses showed that 26 target genes of DEmiRNAs were associated with inositol phosphate metabolism and phosphatidylinositol signalling pathways including INPPL1, INPP5J, INPP5A, INPP5B, INPP4A, INPP4B, INPP1, IMPAD1, DGKZ, CDLPT, CALM1, PLCG1, PLCCD3, PLCB4, PIP4K2A, PIK3R1 and PIK3CB, PIK3CA, ITPKC and ITPK1 genes which may be key genes regulating phosphatidylinositol 4,5-bisphosphate (PIP2), phospholipase C (PLC), inositol triphosphate (IP3), protein kinase C (PKC), calcium (Ca^2+^) and diacylglycerol (DAG).

**Conclusion:**

The findings of this study provide a theoretical framework for a more comprehensive understanding of the biological functions of miRNAs in muscle tissues, and lay a foundation for the discovery and utilisation of high-quality germplasm resources of the Diannan small ear pigs.

## 1 Introduction

MicroRNAs (miRNAs) are endogenous, non-coding RNA molecules found in eukaryotes and viruses, typically ranging from 22 to 23 nucleotides in length. These sequences exhibit a high degree of conservation across species and demonstrate significant temporal and tissue-specific expression patterns (1). The formation of mature miRNAs occurs through the enzymatic action of Dicer, which cleaves double-stranded RNA precursors that are approximately 70 nucleotides long and possess a hairpin structure. In mammalian genomes, miRNAs are implicated in the regulation of mRNA expression for over 30% of genes (2). Studies have proposed that miRNAs play a critical role in modulating various physiological processes, including cell proliferation, differentiation, development, morphogenesis, lipid metabolism, and oncogenesis, primarily through their interactions with mRNA, which includes their influence on the proliferation and differentiation of muscle cells and the proliferation and transformation of muscle fibers during skeletal muscle development (3).

In China, “Big food view” were proposed to reflect the increasing diversification, comprehensiveness, and balance of nutritional needed among the population (4). Pork serves as the primary source of animal protein for human (5). Studies indicated that imported pig breeds exhibit rapid growth rates and high lean meat percentages (6). However, the intramuscular fat content in Chinese local pig breeds is generally higher than that of their imported counterparts, which significantly contributes to the superior meat quality (7). The Diannan small ear pig, recognized as a national breed of livestock genetic resources, is found in the tropical and subtropical regions of southern Yunnan Province. This breed is characterized by its thin skin, small bone structure, and tender, flavorful meat, making it highly desirable among consumers (8). Furthermore, compared to Landrace pigs, Diannan small ear pigs possess a greater intramuscular fat content, rendering them as an ideal model for investigating intramuscular fat deposition (9).

To date, there has been no documented analysis of the miRNAs expression profile in the muscle tissue of Diannan small ear pigs. Yunnan local Diannan Small ear pigs were analyzed, with Landrace pigs (an introduced breed) as the control group. The study aims to employ high-throughput sequencing technology to analyze and predict the differentially expressed miRNAs-specific regulatory target genes, to identify novel miRNAs in both pig breeds, as well as to expand the catalog of candidate miRNAs expressed in porcine muscle tissues.

## 2 Materials and methods

### 2.1 Animals and sample collection

All experimental procedures were conducted in accordance with the Guidelines for the Care and Use of Experimental Animals approved by the Animal Ethics Committee of Yunnan Agricultural University. The animal feeding and sample collection has been detailed by Li et al. (10). Briefly, Diannan small ear pigs and Landrace pigs were raised under the same feeding conditions. When the pigs reached 100 kg body weight, Diannan small ear pigs (DN, *n* = 3) and Landrace pigs (LW, *n* = 3) were slaughtered. Longissimus dorsi muscle samples were collected, quickly frozen and stored in a refrigerator at −80°C.

### 2.2 Extraction and detection of high quality total RNA

Total RNA was extracted from porcine Longissimus dorsi muscle tissue utilizing a conventional method involving Trizol lysis. The extracted RNA was subsequently analyzed through 1% agarose gel electrophoresis to assess the presence of 18S rRNA and 28S rRNA bands, which facilitated the evaluation of RNA degradation and potential contamination. RNA purity was quantified using a NanoDrop spectrophotometer, with a satisfactory OD260/OD280 ratio of ≥1.8. Following the confirmation of the RNA quality of all samples, the construction of a small RNA library was initiated.

### 2.3 Construction and sequencing of small RNA libraries

3 μg of total RNA was taken for library construction. The unique structural characteristics of small RNA, specifically the complete phosphate group at the 5′ end and the hydroxyl group at the 3′ end, facilitated the use of total RNA as the starting material. Adapters were ligated to both ends of small RNAs, followed by reverse transcription into complementary DNA (cDNA). The reaction mixture contained 1 μL random primers, 4 μL 5X First Strand Buffer, 2 μL 100 mM DTT, 0.4 μL 25 mM dNTP Mix, and 0.5 μL RNaseOUT, followed by incubation at 25°C for 2 min. Subsequently, 1 μL SuperScript II Reverse Transcriptase was added, and the reaction was carried out in a PCR thermocycler under the following program: 25°C for 10 min, 42°C for 50 min, 70°C for 15 min, and a final hold at 4°C. RNase-free water, GEX Second Strand Buffer, and 25 mM dNTP Mix were added to the reaction system, followed by incubation on ice for 5 min. RNaseH was then introduced, and the mixture was incubated at 16°C for 2.5 h in the PCR thermocycler. The newly synthesized cDNA strands were blunt-ended using T4 DNA polymerase and Klenow DNA polymerase. A single ‘A’ overhang was added to the 3′ ends of the DNA fragments, followed by ligation of adapters to both termini.

The cDNA underwent amplification via polymerase chain reaction (PCR), and the target DNA fragments were isolated through polyacrylamide gel electrophoresis (PAGE). PCR amplification was performed to enrich the purified cDNA templates. The PCR reaction mixture comprised 10 μL 5X Phusion Buffer, 0.5 μL Phusion DNA Polymerase, 0.5 μL 25 mM dNTP Mix, 1 μL PCR Primer PE 2.0, 7 μL RNase-free H₂O, and 1 μL PCR Primer PE 1.0. Amplification conditions included an initial denaturation at 98°C for 30 s, followed by 15 cycles of 98°C for 10 s, 65°C for 30 s, and 72°C for 30 s, with a final extension at 72°C for 5 min and hold at 4°C. The resulting cDNA library was obtained through gel recovery. Upon completion of the cDNA library construction, initial quantification was conducted using Qubit 2.0, diluting the library to a concentration of 1 ng/μL. The insert size of the library was then assessed using the Agilent 2100 Bioanalyzer. Once the insert size conformed to the anticipated parameters, the effective concentration of the library was accurately determined using quantitative PCR (qPCR), ensuring that the effective library concentration exceeded 2 nM to maintain quality standards. High-throughput sequencing of the small RNA was performed using the Illumina HiSeq 2500/2000 platform.

### 2.4 miRNAs sequence analysis

The raw sequencing data were processed by removing adapter sequences and low-quality reads, yielding clean reads. These clean reads were subsequently aligned with reference sequences utilizing Bowtie. For the small RNA sequences that matched, the software miRBase 20.0 and sRNA-tools-cli were employed to identify both known and potential microRNAs (miRNAs) along with their secondary structures. To ensure unique annotation of each small RNA, this study integrated known miRNAs with RepeatMasker, the Rfam database, and the porcine gene database. Additionally, analyses of non-coding RNAs (ncRNAs), comparisons of repeat sequences, and intron-exon comparisons were conducted, adhering to a hierarchy of priority that included known miRNAs, rRNA, RNA, snRNA, snoRNA, repeat sequences, genes, and novel miRNAs. Coding genes, repetitive sequences, rRNA, tRNA, snRNA, and snoRNA were excluded from the dataset to improve data quality. Predictive analysis of novel miRNAs was carried out by combining the miREvo (11) and miRDeep2 (12) software, focusing on the characteristic hairpin structure, Dicer enzyme cleavage site information, and the energy profiles of miRNAs precursors.

### 2.5 miRNAs expression and differential analysis

In this study, the expression levels of both known and novel microRNAs (miRNAs) in each sample were quantified and subsequently normalized using Transcripts Per Million (TPM) methodology, as defined by the equation: TPM = (read count × 1,000,000) / library size (13). For the analysis of samples that included biological replicates, the DESeq method, which assumes a negative binomial distribution (14). The criterion for identifying differentially expressed miRNAs was set at a false discovery rate (FDR) of less than 0.05. In contrast, for samples lacking biological replicates, the read count data were initially normalized using the Trimmed Mean of M-values (TMM) approach, followed by differential expression analysis utilizing the DEGseq method (15). The screening criteria for differential miRNAs include *q*-value < 0.05 and |log2(fold change)| > 2.

### 2.6 miRNAs target gene prediction and enrichment analysis

The target genes of both known and novel microRNAs (miRNAs) were predicted utilizing the miRanda and TargetScan algorithms, allowing for the identification of relationships between significantly differentially expressed miRNAs (DEmiRNAs) and their corresponding target genes. Subsequently, the target genes associated with the DEmiRNAs were subjected to analysis through Gene Ontology (GO) using Goseq software (16), resulting in the generation of annotated histograms that encompassed cellular components, biological processes, and molecular functions. Additionally, KEGG enrichment analysis was conducted on the aforementioned genes using KOBAS software to elucidate the biological functions of each target gene (17).

## 3 Results

### 3.1 Quality analysis of small RNA sequencing

**Figure 1:**
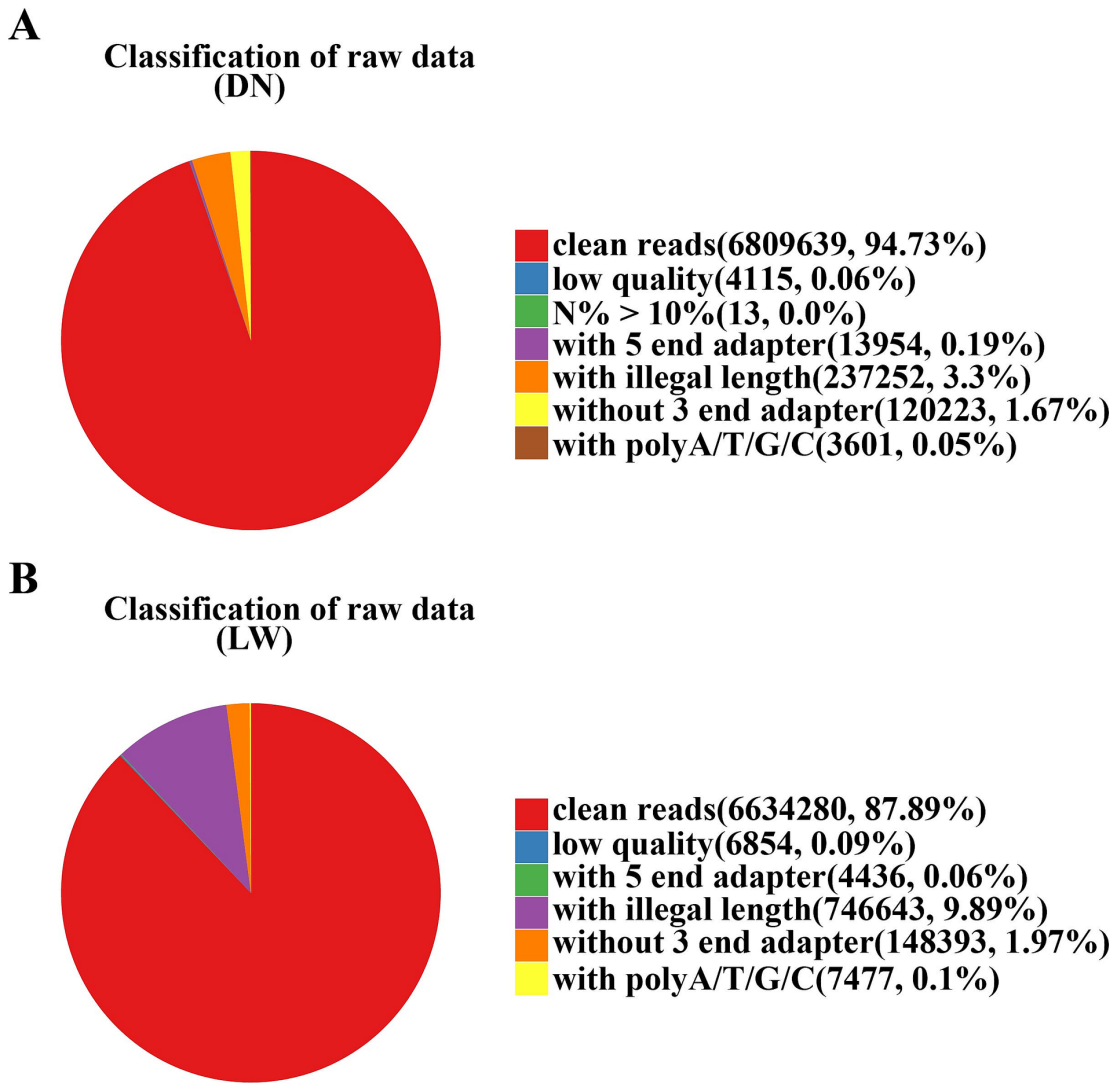
Filter results of raw data. In the analysis of sequencing data, the samples from Diannan small ear pigs and Landrace pigs are denoted by the abbreviations DN and LW, respectively. The filtering results of the raw data are represented in the form of a graph. The red color represents the pure sequence values obtained after processing and their ratio to the original sequence. The other colors represent sequences with N content exceeding 10%, low quality, containing 5 ‘and 3’ adapters, ploy-A/T/G/C tails, and useless sequences with lengths outside of 18–40 bp. **(A)** The raw data analysis results for Diannan Small-ear pigs; **(B)** The raw data analysis results for Landrace pigs.

The filtering results of the raw data are shown in Figure 1. The pure sequence obtained from the DN sample after processing and impurity removal is 6,809,639, accounting for 94.73% of the total number of original sequences; The pure sequence obtained from the LW sample after processing and impurity removal is 6,634,280, accounting for 87.89% of the total original sequence. This indicates that both samples exhibit good sequencing performance and higher sequence quality.

**Figure 2:**
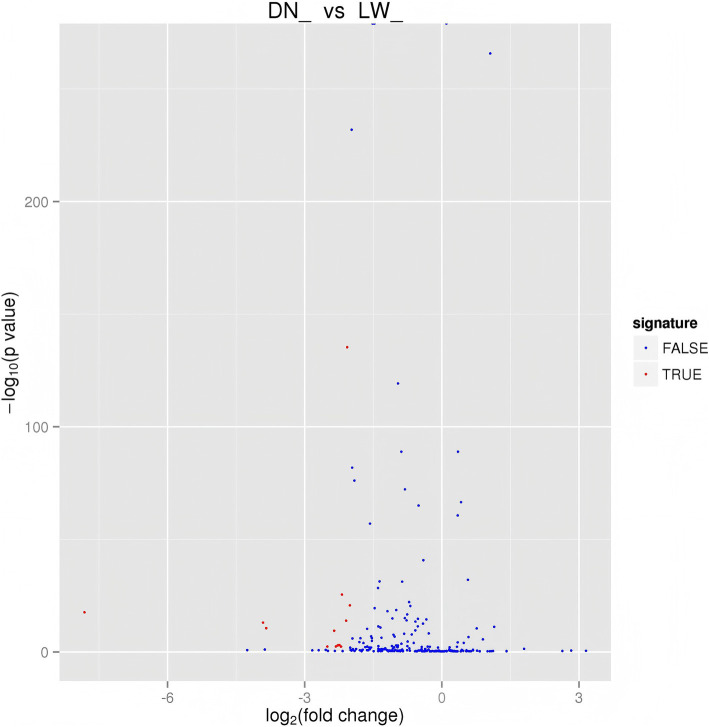
Differential miRNA volcano map. The horizontal axis represents the expression fold changes of miRNA in different experimental groups/samples, and the vertical axis represents the statistical significance of miRNA expression level changes The scattered points in the figure represent each miRNA. The blue dots represent miRNAs with no significant differences, while the red dots represent miRNAs with significant differences.

### 3.2 Identification and detection of miRNAs

The purified small RNA was aligned to the reference genome utilizing Bowtie, and the distribution of small RNA across the genome was subsequently analyzed. Reads mapped to the mentioned reference sequence were compared to a designated range of sequences in miRBase to extract information regarding the small RNAs present in each sample. As illustrated in Table 1, a total of 241 known miRNAs precursors were identified in sample DN, while 251 known miRNAs precursors were identified in sample LW. The characteristic hairpin structure of miRNAs precursors serves as a basis for predicting novel miRNAs. To facilitate this prediction, we employed two miRNAs prediction software tools, miREvo and miRDeep2. Novel miRNAs were predicted based on the characteristic hairpin structures of precursor miRNAs. The analysis yielded predictions of 20 new miRNAs precursors in sample DN and 22 in sample LW. Detailed sequencing information for the novel miRNAs in each sample is available in Supplementary Tables 1, 2.

**Table 1:** Comparison statistics of known miRNAs and novel miRNAs by sample.

Sample	Known miRNAs	Novel miRNAs
DN	241	20
LW	251	22

### 3.3 miRNAs differential expression analysis

The differentially expression levels of known and novel microRNAs (miRNAs) in library basing on the fold change and the significance level (*p*-value) and screening were evaluated. Our analysis shows that there are 14 differentially expressed miRNAs (DEmiRNAs) between DN and LW (Figure 2). As shown in Supplementary Table 3, further analysis revealed that compared to the LW sample library, the expression levels of all identified DEmiRNAs in the DN were significantly reduced and the most significant difference is miR-27a expression among those DEmiRNAs.

### 3.4 Prediction and functional analysis of DEmiRNAs target genes

**Figure 3:**
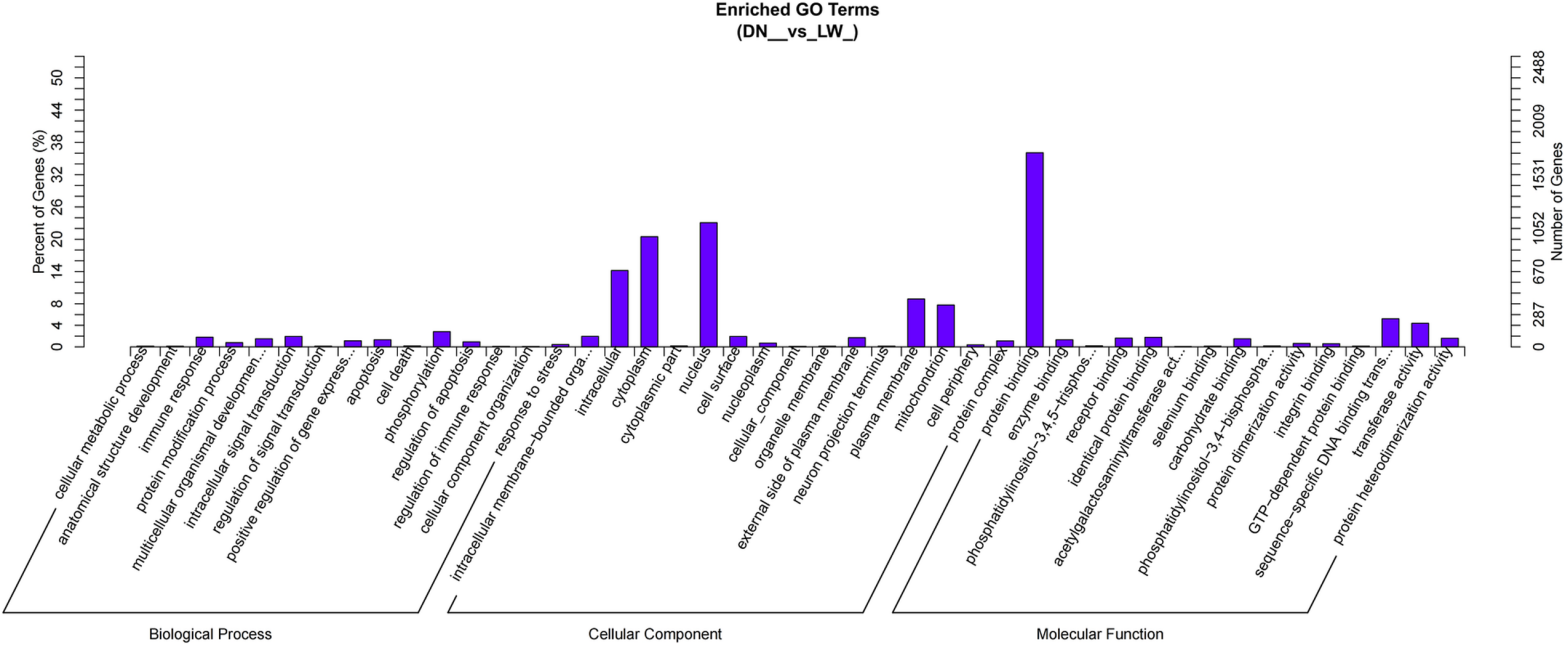
GO enrichment histogram of candidate target genes. The horizontal axis represents the GO term at the next level of the three major categories of GO, and the vertical axis represents the number of candidate target genes annotated under that term (including its sub terms) and their proportion to the total number of annotated candidate target genes. Three different classifications represent the three basic classifications of Go term (from left to right, biological processes, cellular components, and molecular functions).

The target genes of both known and novel microRNAs (miRNAs) in DN and LW samples were predicted using miRanda and TargetScan software. A total of 5,871 target genes associated with 14 differentially expressed miRNAs were identified, resulting in 14,749 mRNA-miRNAs interaction sites. The differentially expressed miRNAs (DEmiRNAs) and their target genes were subsequently analyzed using Gene Ontology (GO) with Goseq software. GO annotation histograms were generated, categorizing the data into cellular components, biological processes, and molecular functions. As illustrated in Figure 3, the candidate target genes exhibited significant enrichment in biological processes related to phosphorylation and intracellular signal transduction, as well as in cellular components such as the nucleus and cytoplasm, and molecular functions associated with protein binding.

**Figure 4:**
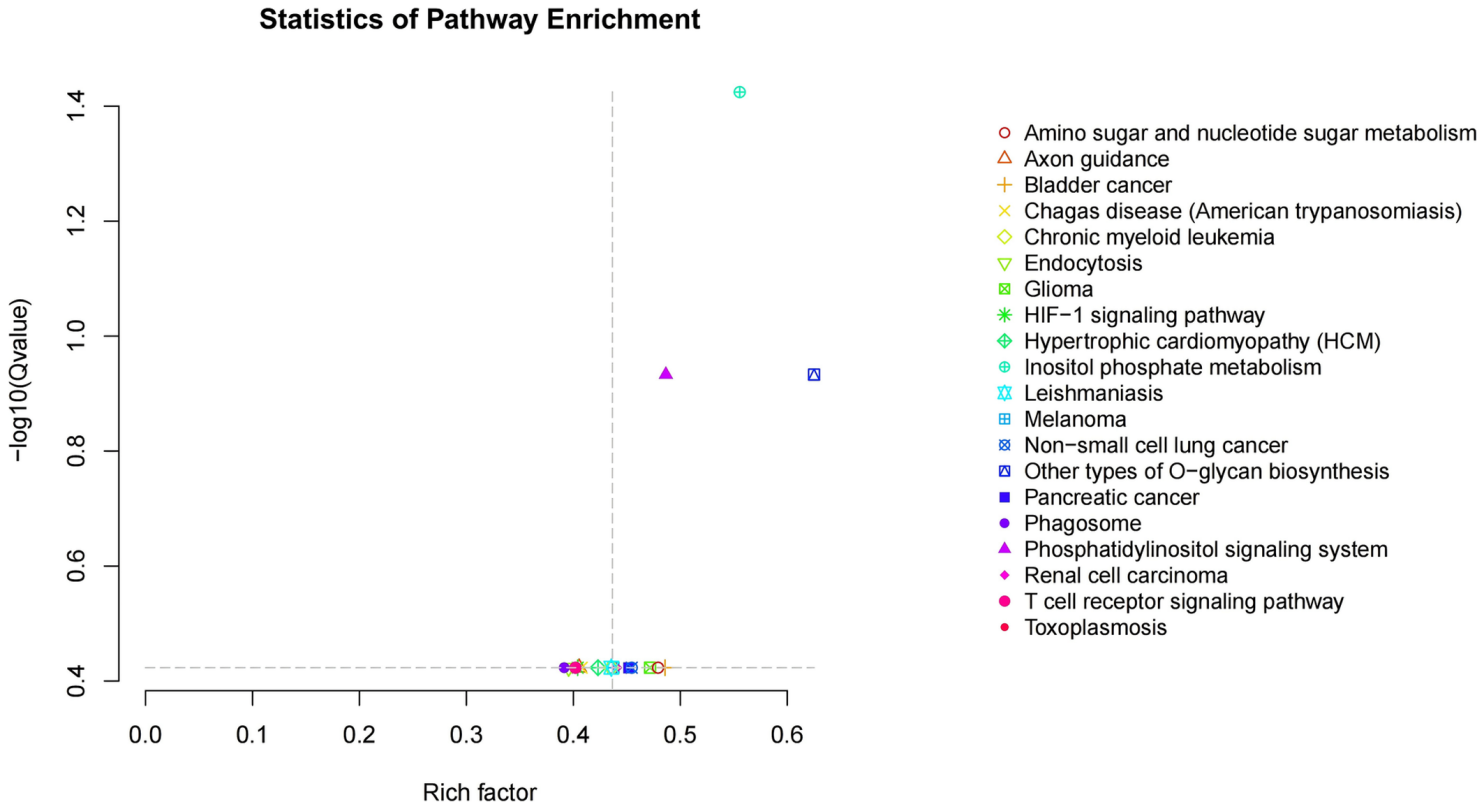
Scatter plot of KEGG enrichment of candidate target genes. Rich factor is the ratio of the number of genes located in the pathway entry among candidate target genes to the number of genes located in the pathway entry among all annotated genes; The larger the Rich factor, the greater the degree of enrichment- The Qvalue in log10Qvalue is the Pvalue after multiple hypothesis testing correction. The larger the - log10Qvalue, the more significant the enrichment.

Kobas software was employed to conduct KEGG enrichment analysis on the aforementioned target genes in order to predict their biological functions. The analysis revealed a total of 248 pathways associated with the miRNAs target genes, of which 20 pathways exhibited significant enrichment (*p*-value < 0.05). As illustrated in Figure 4, the miRNAs target genes demonstrated notable enrichment in pathways such as inositol phosphate metabolism, the phosphatidylinositol signaling system, endocytosis, and various other signaling pathways, with inositol phosphate metabolism identified as the most significantly enriched term. The Inositol phosphate metabolism pathway is a component of the phosphatidylinositol signaling pathway.

### 3.5 Analysis of differentially expressed miRNAs target genes involved in inositol phosphate metabolism

**Figure 5:**
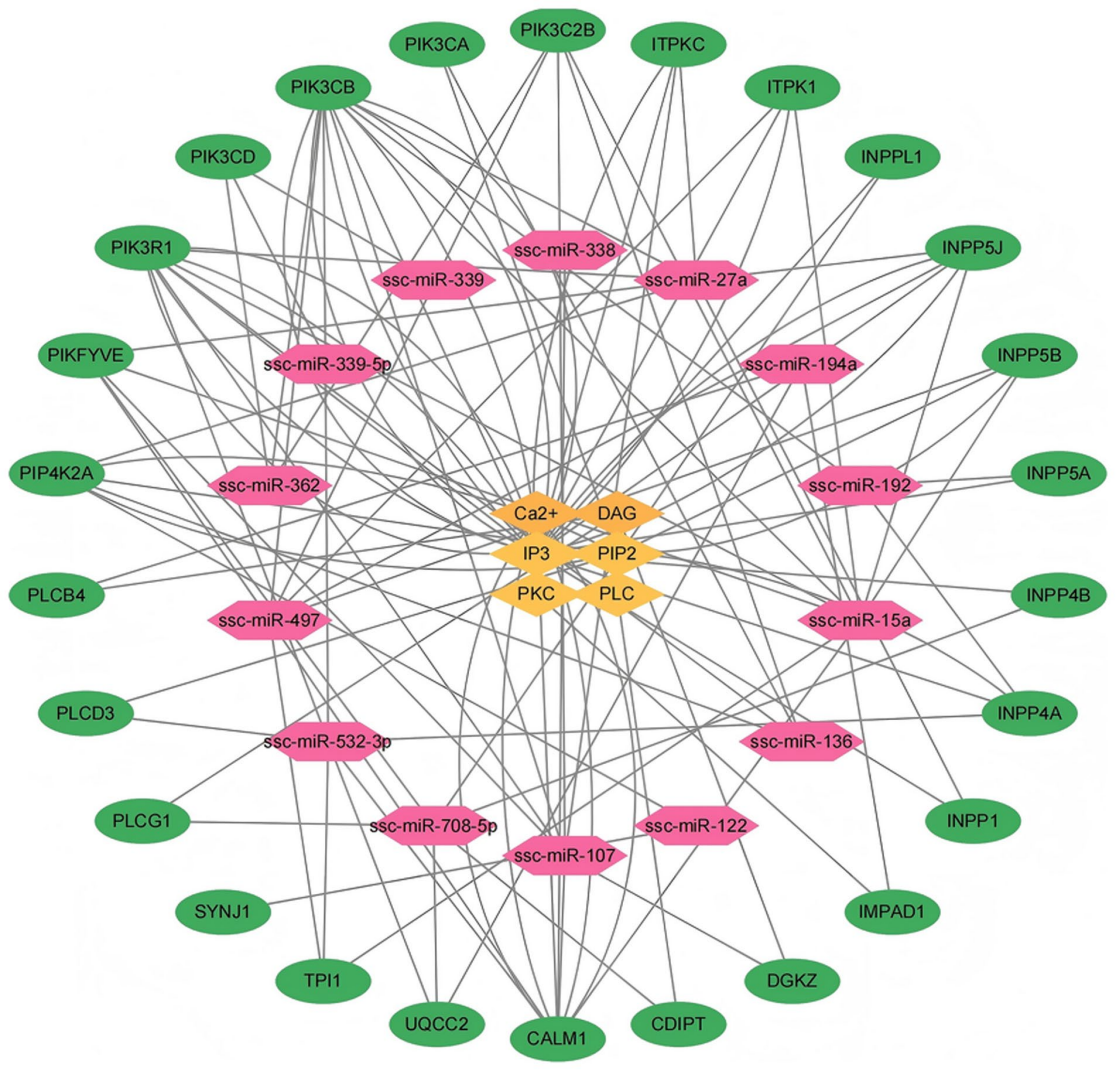
Network diagram of interactions between differentially expressed microRNAs, target genes, and phosphoinositol metabolism regulators. (1) Red represents microRNAs with significant differential expression between two pig breeds. (2) Green indicates the target genes corresponding to significantly differentially expressed microRNAs in phosphoinositol metabolism. (3) Yellow represents the relevant regulatory factors in phosphoinositol metabolism.

In 14 DEmiRNA from DN and LW pigs were identified, which are associated with 26 target genes involved in inositol phosphate metabolism (Supplementary Table 4). The identified target genes may influence myoblast proliferation and differentiation through their role in the regulation of Inositol phosphate metabolism. Figure 5 illustrates the differentially expressed miRNAs associated with Inositol phosphate metabolism alongside their respective target genes.

Specifically, miRNAs-15a is linked to nine target genes including ITPK1, TPI1, INPP5B, PIK3C2B, PIKFYVE, PIK3R1, CALM1, PIK3CB, and INPP5J. miRNAs-497 is associated with eight target genes, which include TPI1, ITPK1, INPP5B, PIK3C2B, PIKFYVE, CALM1, PIK3CB, and INPP5J. miRNAs-27a corresponds to seven target genes including INPP1, PIKFYVE, PIK3R1, INPP5J, ITPKC, PIP4K2A, and PIK3CB. Similarly, miRNAs-107 is linked to seven target genes including DGKZ, ITPKC, PIK3C2B, PIKFYVE, PIK3R1, CALM1, and PIK3CB. miRNAs -532-3p has 7 target genes, namely PIK3CB, PLCD3, CALM1, TPI1, INPP4A, PIK3R1, and UQCC2. miRNAs-708-5p is associated with six target genes including PLCG1, INPP4B, INPPL1, CDIPT, PIKFYVE, and UQCC2. Additionally, miRNAs-136 corresponds to four target genes including PIK3C2B, PIK3CA, PIP4K2A, and PIK3R1. The target genes for miRNA-192 include INPP4A, INPP5A, and PIK3CB, while miRNAs-194a is associated with IMPAD1, PLCB4, and UQCC2 genes. miRNAs-362 corresponds to three target genes including PIK3CD, PIK3C2B, and PIK3CB. The target genes for miRNAs-122 are PIP4K2A and SYNJ1, and the target gene for miRNAs-338 is CALM1. Lastly, both miRNAs-339 and miRNA-339-5p correspond to the target gene PIK3CD.

As shown in Figure 5, the 26 genes including PIP4K2A, PIKFYVE, PIK3R1, PIK3CD, PIK3CB, PIK3CA, PIK3C2B, ITPKC, ITPK1, INPPL1, INPP5J, INP P5B, INPP5A, INPP4B, INPP4A, INPP1, IMPAD1, DGKZ, CDIPT, CALM1, UQCC2, TPI1, SYNJ1, PLCG1, PLCD3 and PLCB4, may be the key genes involved in the regulation of Ca^2+^, DAG, IP3, PIP2, PKC and PLC which participated in the Inositol phosphate metabolism pathway.

## 4 Discussion

### 4.1 miRNAs research based on high-throughput sequencing technology

High-throughput sequencing technology offers numerous advantages, including scalability, efficiency, rapid processing, sensitivity, and cost-effectiveness, which have facilitated the successful identification and characterization of novel microRNAs (miRNAs) in various animal test samples. For instance, Morin et al. (16) employed Solexa sequencing technology to investigate the miRNAs profiles of human embryonic stem cells prior to differentiation, resulting in the identification of a total of 438 miRNAs, of which 104 were newly discovered. Similarly, Chen et al. (17) utilized Solexa high-throughput technology to detect and analyze 113 miRNAs in snail specimens. The role of miRNAs in skeletal muscle growth and development has been well-documented (18, 19). Jiang et al. (20) identified 63 differentially expressed miRNAs between the biceps femoris and soleus muscles in pigs, highlighting their potential regulatory function in skeletal muscle fiber type differentiation. Furthermore, miRNAs are implicated in the regulation of various biological processes, including cellular lipid synthesis and metabolism (21). Li et al. (22) reported the detection of 227 known conserved miRNAs and the identification of 66 novel miRNAs in porcine adipose tissue through the application of Solexa high-throughput technology. These findings underscore the capability of high-throughput sequencing to reveal a substantial number of novel miRNAs. Implementation of high-throughput sequencing technology has significantly enhanced the efficiency and speed of miRNAs candidate gene discovery in mammalian (23).

In this investigation, we identified a total of 241 known conserved microRNAs (miRNAs) and 20 novel candidate miRNAs from the longissimus dorsi muscle of Diannan small ear pigs utilizing high-throughput sequencing technology. Additionally, 251 known conserved miRNAs and 21 new candidate miRNAs were detected in the longissimus dorsi muscle of Landrace pigs. A comparative analysis revealed 14 distinct miRNAs exhibiting significant differential expression between Diannan small ear pigs and Landrace pig. Notably, the expression levels of these 14 miRNAs were markedly lower in Diannan small ear pigs compared to Landrace pigs, and with miR-27a exhibits the most significant difference between two breed pigs (24, 25). Yu et al. (26) propose that miR-27a derived from adipose tissue may play a pivotal role in the development of obesity-induced insulin resistance in skeletal muscle. Furthermore, miR-27a has been shown to inhibit lipid synthesis and adipocyte differentiation by downregulating PPARγ (27). The insulin sensitivity of PPAR was upregulated by miR-27a, which was accompanied by the inhibition of PPARγ expression and increased levels of AKT phosphorylation and GLUT4 (28). miR-27a drives metabolic dysregulation by suppressing PPARα expression in white adipose tissue, leading to elevated free fatty acids and dyslipidemia, while PPARα activation partially reverses these alterations, highlighting its pivotal role in obesity-associated exosomal miRNA-induced glucose intolerance and lipid metabolism dysfunction (21). miR-27a exacerbates obesity-associated inflammation and metabolic dysfunction by suppressing PPARγ, driving M1 polarization and NF-κB-mediated insulin resistance (29). In conclusion, we hypothesize that the observed differences in muscle growth and fat deposition between Diannan small ear pigs and Landrace pigs are associated with the significant differential expression of miR-27a in these two breeds.

### 4.2 miRNAs target gene prediction and functional enrichment analysis

Currently, there exists a variety of software tools designed to predict target genes, among which miRanda and TargetScan are widely utilized. The miRanda software, developed by Enright et al. (30) was the first of its kind for predicting miRNAs target genes. It imposes stringent criteria regarding the evolutionary conservation of miRNAs-target gene interaction sites, which can lead to a high rate of false positives in target gene identification (30). In contrast, TargetScan, developed by Agarwal et al. (2015) assesses target gene predictions by considering the thermodynamic stability and the miRanda tends to generate a high number of predicted targets, which may lead to increased noise in the results (31).

In the present study, we employed both TargetScan and miRanda to analyze and predict 14 differentially expressed miRNAs target gene loci in samples from Diannan small ear pigs and Landrace pigs. Gene Ontology (GO) analysis revealed that the target genes of differentially expressed miRNAs (DEmiRNAs) in both breeds were significantly enriched in biological processes such as immune response and protein modification, as well as in cellular components including the nucleus, cytoplasm, and intracellular regions, and in molecular functions related to protein binding. Furthermore, Kyoto Encyclopedia of Genes and Genomes (KEGG) pathway enrichment analysis indicated that Inositol phosphate metabolism was the most significantly enriched pathway associated with the target genes of DEmiRNAs. We identified 26 target genes that play a role in regulating Inositol phosphate metabolism, which in turn influences the proliferation and differentiation of muscle cells.

### 4.3 DE miRNAs target genes are involved in the regulation of phosphatidylinositol signaling pathway

Inositol phosphates (IPs) represent a category of monophosphate or polyphosphory lated inositols that are integral to a multitude of cellular functions, including cell growth, apoptosis, migration, endocytosis, and differentiation (32). Notable IP species include IP, IP2, IP3, IP4, IP5, and IP6 (33). Inositol phosphates can function as cofactors or as intermolecular facilitators that promote the assembly of proteins, thereby activating various biological processes such as RNA editing (31), RNA export (32), mRNA transcription (34), DNA double-stranded break repair (35, 36), gene expression (37, 38), proteasomal activity (35), and phosphate Homeostasis (39). The role of inositol phosphates in cellular secretion, muscle contraction, and cell proliferation and differentiation is meticulously regulated by a network of genes, many of which are modulated by their associated microRNAs (miRNAs) (40). To investigate the contribution of these genes to inositol phosphate metabolism, this study identified 14 differentially expressed miRNAs that are enriched in target genes related to inositol phosphate metabolism.

As illustrated in Figure 5, the results showed INPP5B, PIP4K2A, PLCD3 and PIK3CA genes are implicated in the regulation of phosphatidylinositol diphosphate (PIP2) with in the phosphatidylinositol signaling pathway. Phosphatidylinositol phosphatase (INPP5B) deficiency affects spermatogenesis and maturation, leading to infertility (41). PIP4K2A plays a critical role in intracellular cholesterol transport by upregulating the levels of PI (4, 5) P2 in the peroxisome membrane (42). Phospholipase C Delta 3 (PLCD3) is a member of phospholipase C (PLC) protein, which promotes cell proliferation, migration, and invasion through PI3K/AKT/P21 signaling (43). PIK3CA encodes the catalytic subunit p110 alpha of PI3K (44). PIK3CA is involved in the mTOR and PI3K Akt signaling pathways, and studies have shown that lipid metabolism is regulated by the PI3K Akt mTOR signaling pathway (45).

Additionally, three genes, namely DGKZ, PLCB4, and PLCG1, are involved in the regulation of diacylglycerol (DAG). Diacylglycerol kinase (DGK) is involved in lipid mediated signal transduction. The ratio of Phosphorylates diacylglycerol (DG) to phosphatidic acid (PA) regulates the balance and control of these second messenger actions (46). Diacylglycerol kinase zeta (DGKZ) is associated with the pathogenesis of various malignant diseases (47). Downregulation of DGKZ can hinder cell proliferation, promote cell apoptosis, and induce cell cycle arrest, thereby inhibiting the occurrence and progression of cervical cancer tumors (48). PLCB4 encodes PLC *β* 4 protein, which is one of the subtypes of phospholipase C (PLC) (49). The PLCG1 gene encodes the phospholipase C *γ* 1 subtype (50). Phospholipase C (PLC) γ 1 is a key enzyme that regulates nuclear factor kappa B (NF - *κ* B), extracellular signal related kinases, mitogen activated protein kinases, and activated T cell signaling pathways (51). PLCs catalyze PtdIns (4, 5) P2 to form two intracellular second messengers, namely diacylglycerol (DAG) and inositol 4,5,3-triphosphate (InsP3), which play important roles in signal transduction (52).

Furthermore, 10 genes contribute to the regulation of inositol triphosphate (IP3), which include INPP4A, INPP4B, INPP5A, INPP5J, INPP1, INPPL1, ITPKC, PIK3CB, PIK3R1 and CDIPT. Type I and type II 4-phosphatases (INPP4A and INPP4B) are the only known PtdIns(3,4)P2 phosphatases, and they are homologous within their catalytic domain with P-Rex proteins (53). Inositol polyphosphate 5-phosphatase (INPP5A) belongs to the large family of inositol polyphosphate 5-phosphatase. As an intracellular calcium mobilizer and modifier enzyme, it can promote cellular responses to various stimuli (54). Research has found that INPP5J is the main target molecule regulating demyelination through glycerolipid and glycerophospholipid metabolism, phosphatidylinositol signaling, and estrogen signaling in pre demyelinating forebrain slice cultures (FSC) (55). The inositol polyphosphate 1-phosphatase gene (INPP1) catalyzes the hydrolysis of inositol 1,3,4-triphosphate and inositol 1,4-diphosphate, with inositol 42,49-diphosphate being a key molecule in phosphoinositide metabolism and signaling pathways (56). The position candidate gene INPPL1 has been discovered in Landrace pigs, which has a negative regulatory effect on diet induced obesity and participates in the regulation of insulin function (57). The enzyme encoded by ITPKC can catalyze the phosphorylation of inositol 1,4,5-triphosphate to 1,3,4,5-tetraphosphate (57). *In vitro* functional analysis using luciferase assay showed that ITPKC mutations may reduce the splicing efficiency of its mRNA expression level (58). PIK3CB encodes the catalytic subunit p110beta of PI3K (59). The intracellular lipid accumulation, mRNA expression, and protein content of genes related to *de novo* fatty acid synthesis are all regulated by the PI3K Akt mTOR pathway (60). Phosphoinositide-3-kinase regulatory subunit 1(PIK3R1) was identified as promising candidate genes for milk production traits due to their being differentially expressed between the dry period and the peak of lactation in livers of dairy cows (61). The CDIPT is crucial to the fatty acid metabolic pathway, intracellular signal transduction and energy metabolism in eukaryotic cells (62).

The gene that regulates the signaling pathway related to intracellular calcium ion (Ca^2+^) concentration is CALM1. CALM1 mainly participates in the regulation of boar sperm motility through the cAMP/PKA signaling pathway, indicating that protein phosphorylation may be an important mechanism affecting sperm diversity (63).

The gene IMPAD1 is responsible for regulating protein kinase C (PKC) within the signaling pathway, while CDIPT modulates the activity of phospholipase C (PLC) in this context. Upstream mediators such as PLAG1, IMPAD1, and TUFM can regulate AMPK-mediated metastasis (64). In the phosphatidylinositol metabolic pathway, extracellular signaling molecules interact with G protein-coupled receptors located on the cell surface, which subsequently activate phospholipase C (PLC-*β*) on the plasma membrane. This activation leads to the hydrolysis of phosphatidylinositol 4,5-bisphosphate (PIP2) into 2 s messengers: inositol 1,4,5-trisphosphate (IP3) and diacylglycerol (DAG) (65). This process facilitates the conversion of extracellular signals into intracellular signals. IP3 binds to ligand-gated calcium channels on the endoplasmic reticulum, resulting in the opening of these channels and an increase in intracellular Ca2 + concentration, which in turn activates various calcium-dependent proteins (66). DAG, when bound to the plasma membrane, activates PKC, which is initially present in the cytosol in an inactive form (67). Upon stimulation of the cell, the production of IP3 leads to an increase in Ca^2+^ concentration, prompting PKC to translocate to the inner surface of the plasma membrane where it is activated by DAG (68). PKC is capable of phosphorylating serine/threonine residues on proteins, eliciting diverse cellular responses such as secretion, muscle contraction, and cell proliferation and differentiation, which vary depending on the specific cell type involved.

## 5 Conclusion

A total of 14 differentially expressed microRNAs (miRNAs) were identified within the small RNA library derived from the latissimus dorsi muscle of Diannan small ear pigs and Landrace pigs. 26 target genes associated with these differentially expressed miRNAs were involved in inositol phosphate metabolism and phosphatidylinositol signalling pathways. The further studies are needed to confirm the function of differentially expressed microRNAs (miRNAs) and target genes in the muscle tissue of pigs.

## Data Availability

Sequence data that support the findings of this study have been deposited in the NCBI database with the accession number PRJNA1279147.
