# Supplementary material for: MicroRNA analysis of porcine muscle tissue involved in phosphoinositol metabolism
Source: Front Vet Sci. 2025 Jul 23;12:1482031. doi: 10.3389/fvets.2025.1482031 (PMC12329466; doi:10.3389/fvets.2025.1482031)
Supplement: Supplementary file 1 [file Table_1.DOCX]

Supplementary Material

**Table S1|** Sequence information of novel miRNAs in DN samples

| Numbering of novel miRNAs(DN) | Sequence information |
| --- | --- |
| ssc.novel 1 | ccgaccucccguagcucacuu |
| ssc.novel 2 | acugaauggaauugucucagcc |
| ssc.novel 3 | acugaauggaauugucucagcc |
| ssc.novel 4 | agucuugauucucuguuuugu |
| ssc.novel 6 | cgucucucugcuagggguucugu |
| ssc.novel 7 | uuguccgugccccacccacuca |
| ssc.novel 9 | uuggcucugcgaggucggcuca |
| ssc.novel 12 | uuggcucugcgaggucggcuca |
| ssc.novel 13 | aaaugaaaaggauugguuucuc |
| ssc.novel 14 | uuuguuggcuccucugaaguga |
| ssc.novel 15 | cuugcccgagagcuuggaccgc |
| ssc.novel 16 | caacgugcugacugcggcaga |
| ssc.novel 17 | auucuguuagaaaaaugcaaga |
| ssc.novel 18 | gucccaucugggucgcca |
| ssc.novel 19 | gagggcaagucuggugcc |
| ssc.novel 20 | agacugaagcuccuugaga |
| ssc.novel 22 | uuugcucugcuccugccacaugc |
| ssc.novel 23 | gcaauuuagguuaaugacuguc |
| ssc.novel 24 | uuugcucugcuccugccacaugc |
| ssc.novel 25 | cuugcccgagagcuuggaccgc |

**Table S2|** Sequence information of novel miRNAs in LW samples

| Numbering of novel miRNAs(LW) | Sequencing sequence information |
| --- | --- |
| ssc.novel 1 | ccgaccucccguagcucacuu |
| ssc.novel 2 | acugaauggaauugucucagcc |
| ssc.novel 3 | acugaauggaauugucucagcc |
| ssc.novel 5 | uuuuaacagggaguucccaucac |
| ssc.novel 6 | cgucucucugcuagggguucugu |
| ssc.novel 7 | uuguccgugccccacccacuca |
| ssc.novel 9 | uuggcucugcgaggucggcuca |
| ssc.novel 10 | gaagagucacugucugaaagaagca |
| ssc.novel 12 | uuggcucugcgaggucggcuca |
| ssc.novel 13 | aaaugaaaaggauugguuucuc |
| ssc.novel 14 | uuuguuggcuccucugaaguga |
| ssc.novel 15 | cuugcccgagagcuuggaccgc |
| ssc.novel 16 | caacgugcugacugcggcaga |
| ssc.novel 17 | auucuguuagaaaaaugcaaga |
| ssc.novel 18 | gucccaucugggucgcca |
| ssc.novel 19 | gagggcaagucuggugcc |
| ssc.novel 20 | agacugaagcuccuugaga |
| ssc.novel 21 | agauugugaaucuagaca |
| ssc.novel 22 | uuugcucugcuccugccacaugc |
| ssc.novel 23 | gcaauuuagguuaaugacuguc |
| ssc.novel 24 | uuugcucugcuccugccacaugc |
| ssc.novel 25 | cuugcccgagagcuuggaccgc |

**Table S3|** Analysisof miRNA differentialexpression

| miRNA | DN | LW | log2(Fold change) normalized | q-value |
| --- | --- | --- | --- | --- |
| ssc-miR-107 | 36 | 155 | -2.276667288 | 0.001091449 |
| ssc-miR-122 | 3 | 601 | -7.816726564 | 2.48E-18 |
| ssc-miR-136 | 40 | 168 | -2.240857212 | 0.000762089 |
| ssc-miR-15a | 400 | 1434 | -2.012441003 | 1.99E-21 |
| ssc-miR-192 | 34 | 454 | -3.90955353 | 8.93E-14 |
| ssc-miR-194a | 29 | 369 | -3.839963895 | 2.61E-11 |
| ssc-miR-27a | 2459 | 9175 | -2.070104307 | 4.70E-136 |
| ssc-miR-338 | 28 | 124 | -2.317309272 | 0.003332471 |
| ssc-miR-339 | 396 | 1598 | -2.183162957 | 3.04E-26 |
| ssc-miR-339-5p | 396 | 1598 | -2.183162957 | 3.04E-26 |
| ssc-miR-362 | 237 | 899 | -2.093901941 | 1.35E-14 |
| ssc-miR-497 | 114 | 518 | -2.354386158 | 3.88E-10 |
| ssc-miR-532-3p | 22 | 111 | -2.505452132 | 0.003648844 |
| ssc-miR-708-5p | 31 | 127 | -2.20495626 | 0.003888387 |

**Table S4|** Target genes related to the phosphoinositide metabolism

| miRNA | Target genes related to the phosphoinositide metabolism |
| --- | --- |
| ssc-miR-15a | Inositol-Tetrakisphosphate 1-Kinas (ITPK1)  Triosephosphate Isomerase (TPI1)  nositol polyphosphate-5-phosphatase, 75kDa (INPP5B) Phosphatidylinositol-4-Phosphate 3-Kinase (PIK3C2B) Phosphoinositide Kinase (PIKFYVE) Phosphoinositide-3-Kinase, Regulatory Subunit 1 (Alpha) (PIK3R1)  Calmodulin 1(CALM1 ) Phosphatidylinositol-4,5-Bisphosphate 3-Kinase (PIK3CB) Inositol Polyphosphate-5-Phosphatase J(INPP5J) |
| ssc-miR-27a | Inositol Polyphosphate-1-Phosphatase (INPP1) Phosphoinositide Kinase (PIKFYVE) Phosphoinositide-3-Kinase, Regulatory Subunit 1(PIK3R1)  Inositol Polyphosphate-5-Phosphatase J(INPP5J) Inositol-Trisphosphate 3-Kinase C (ITPKC ) Phosphatidylinositol-5-Phosphate 4-Kinase, Type II, Alpha (PIP4K2A)  Phosphatidylinositol-4,5-Bisphosphate 3-Kinase (PIK3CB) |
| ssc-miR-107 | Diacylglycerol Kinase, Zeta (DGKZ)  Inositol-Trisphosphate 3-Kinase C (ITPKC ) Phosphatidylinositol-4-Phosphate 3-Kinase (PIK3C2B) Phosphoinositide Kinase (PIKFYVE) Phosphoinositide-3-Kinase, Regulatory Subunit 1(PIK3R1)  Calmodulin 1(CALM1 ) Phosphatidylinositol-4,5-Bisphosphate 3-Kinase (PIK3CB) |
| ssc-miR-122 | Phosphatidylinositol-5-Phosphate 4-Kinase, Type II, Alpha(PIP4K2A)  Synaptojanin 1(SYNJ1) |
| ssc-miR-136 | Phosphatidylinositol-4-Phosphate 3-Kinase (PIK3C2B) Phosphatidylinositol-4,5-Bisphosphate 3-Kinase, Catalytic Subunit Alpha(PIK3CA) Phosphatidylinositol-5-Phosphate 4-Kinase, Type II, Alpha(PIP4K2A)  Phosphoinositide-3-Kinase, Regulatory Subunit 1(PIK3R1) |
| ssc-miR-192 | Inositol polyphosphate-4-phosphatase, type I(INPP4A) Inositol polyphosphate-5-phosphatase, 40kDa(INPP5A) Phosphatidylinositol-4,5-Bisphosphate 3-Kinase (PIK3CB) |
| ssc-miR-194a | Inositol Monophosphatase Domain Containing 1(IMPAD1) Phospholipase C, Beta 4(PLCB4) Ubiquinol-Cytochrome C Reductase Complex Assembly Factor (UQCC2) |
| ssc-miR-338 | Calmodulin 1(CALM1 ) |
| ssc-miR-339 | Phosphatidylinositol-4,5-Bisphosphate 3-Kinase( PIK3CD) |
| ssc-miR-339-5p | Phosphatidylinositol-4,5-Bisphosphate 3-Kinase( PIK3CD) |
| ssc-miR-362 | Phosphatidylinositol-4,5-Bisphosphate 3-Kinase( PIK3CD) Phosphatidylinositol-4-Phosphate 3-Kinase (PIK3C2B) Phosphatidylinositol-4,5-Bisphosphate 3-Kinase (PIK3CB) |
| ssc-miR-497 | Triosephosphate Isomerase (TPI1) Inositol-Tetrakisphosphate 1-Kinas (ITPK1)  inositol polyphosphate-5-phosphatase, 75kDa (INPP5B) Phosphatidylinositol-4-Phosphate 3-Kinase (PIK3C2B) Phosphoinositide Kinase (PIKFYVE) Calmodulin 1(CALM1) Phosphatidylinositol-4,5-Bisphosphate 3-Kinase (PIK3CB) Inositol Polyphosphate-5-Phosphatase J(INPP5J) |
| ssc-miR-532-3p | Phosphatidylinositol-4,5-Bisphosphate 3-Kinase (PIK3CB) Phospholipase C, Delta 3(PLCD3) Calmodulin 1(CALM1 ) Triosephosphate Isomerase (TPI1) inositol polyphosphate-4-phosphatase, type I(INPP4A) Phosphoinositide-3-Kinase, Regulatory Subunit 1 (Alpha) (PIK3R1)  Ubiquinol-Cytochrome C Reductase Complex Assembly Factor (UQCC2) |
| ssc-miR-708-5p | Phospholipase C, Gamma (PLCG1) inositol polyphosphate-4-phosphatase, type II, 105kDa(INPP4B) Inositol Polyphosphate Phosphatase-Like 1(INPPL1) CDP-Diacylglycerol--Inositol 3-Phosphatidyltransferase (CDIPT) Phosphoinositide Kinase (PIKFYVE) Ubiquinol-Cytochrome C Reductase Complex Assembly Factor (UQCC2) |
